# Supplementary figures and images for: Triple negative breast cancer cells acquire lymphocyte proteins and genomic DNA during trogocytosis with T cells
Source: PeerJ. 2025 Mar 31;13:e19236. doi: 10.7717/peerj.19236 (PMC11967428; doi:10.7717/peerj.19236)

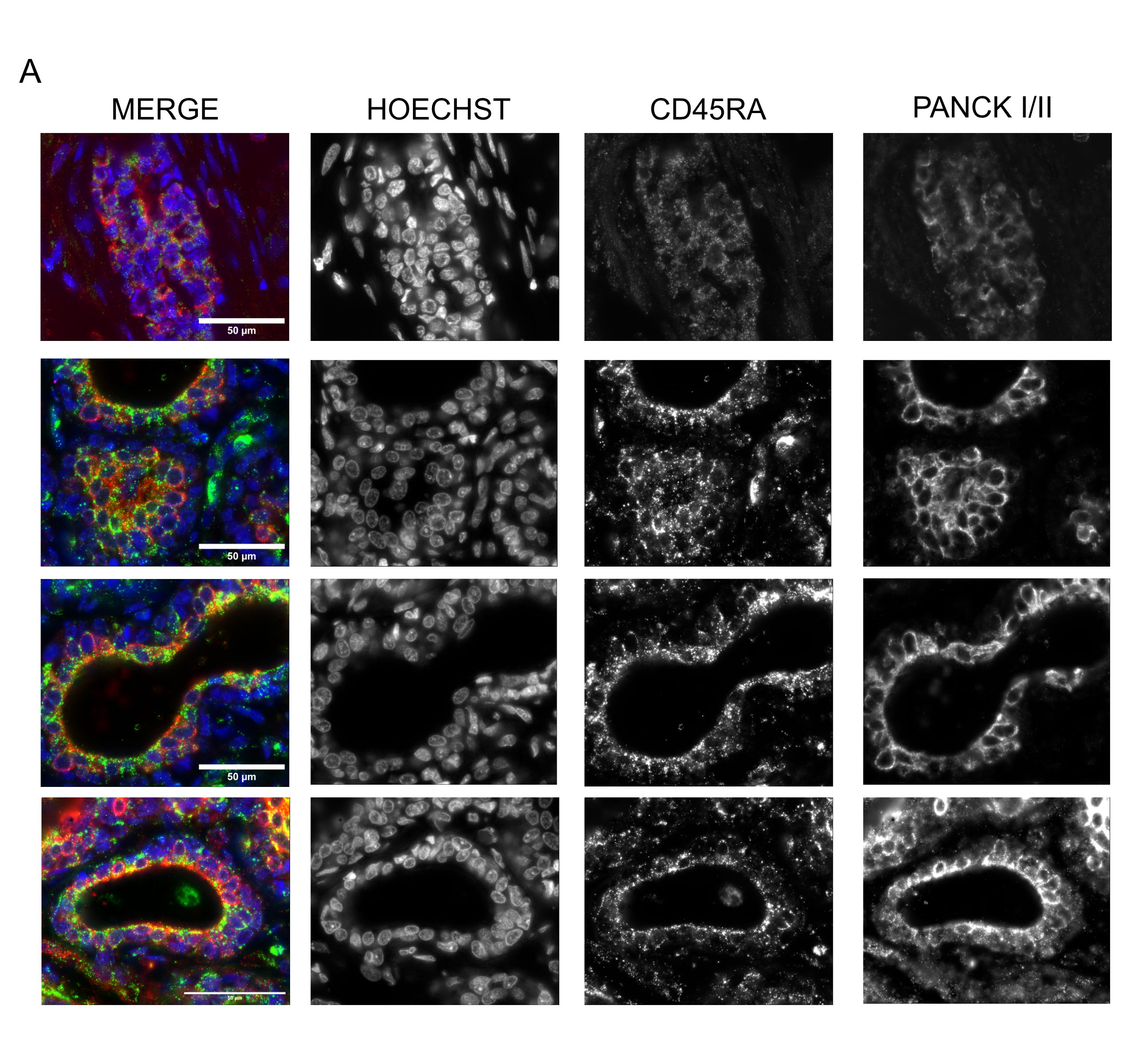

Supplement: Supplemental Information 1 — (A) Merge images are shown on the left panel with single channel images for CD45RA (green), Hoechst nuclear dye (blue), and tumor marker Pan-Cytokeratin Type I/II (red) shown in their respective columns. [file peerj-13-19236-s001.png]

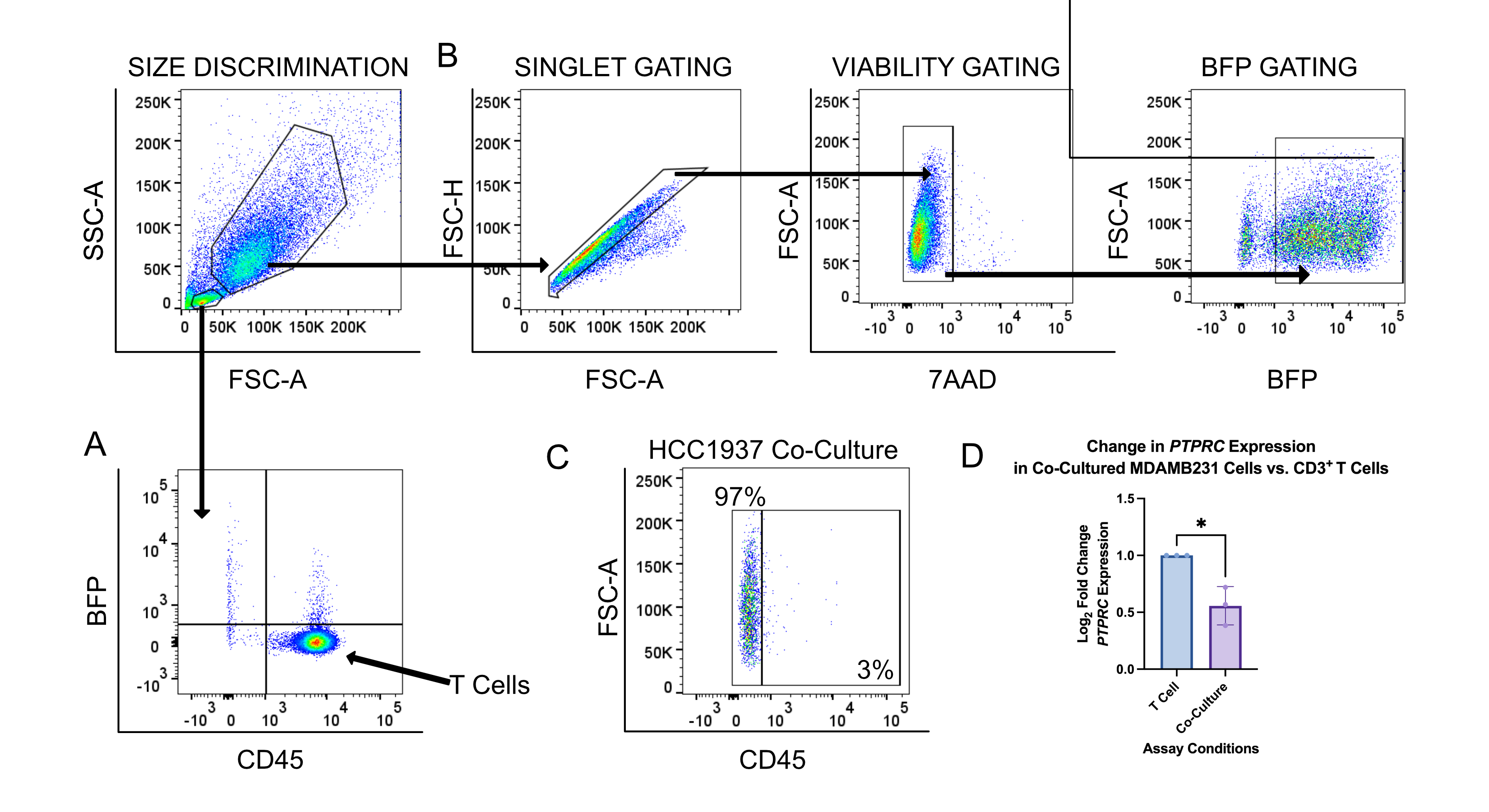

Supplement: Supplemental Information 2 — (A) Analysis of T cells to visualize lack of BFP-LA acquisition post co-culture. (B) Gating strategy to identify viable, BFP-LA + tumor singlets. (C) Co-culture analysis of HCC1937 TNBC cells (D) Change in expression of PTPRC between co-cultured MDAMB231 and primary T cells. An unpaired T test was used to perform statistical analysis on this data *p = 0.0104. [file peerj-13-19236-s002.png]

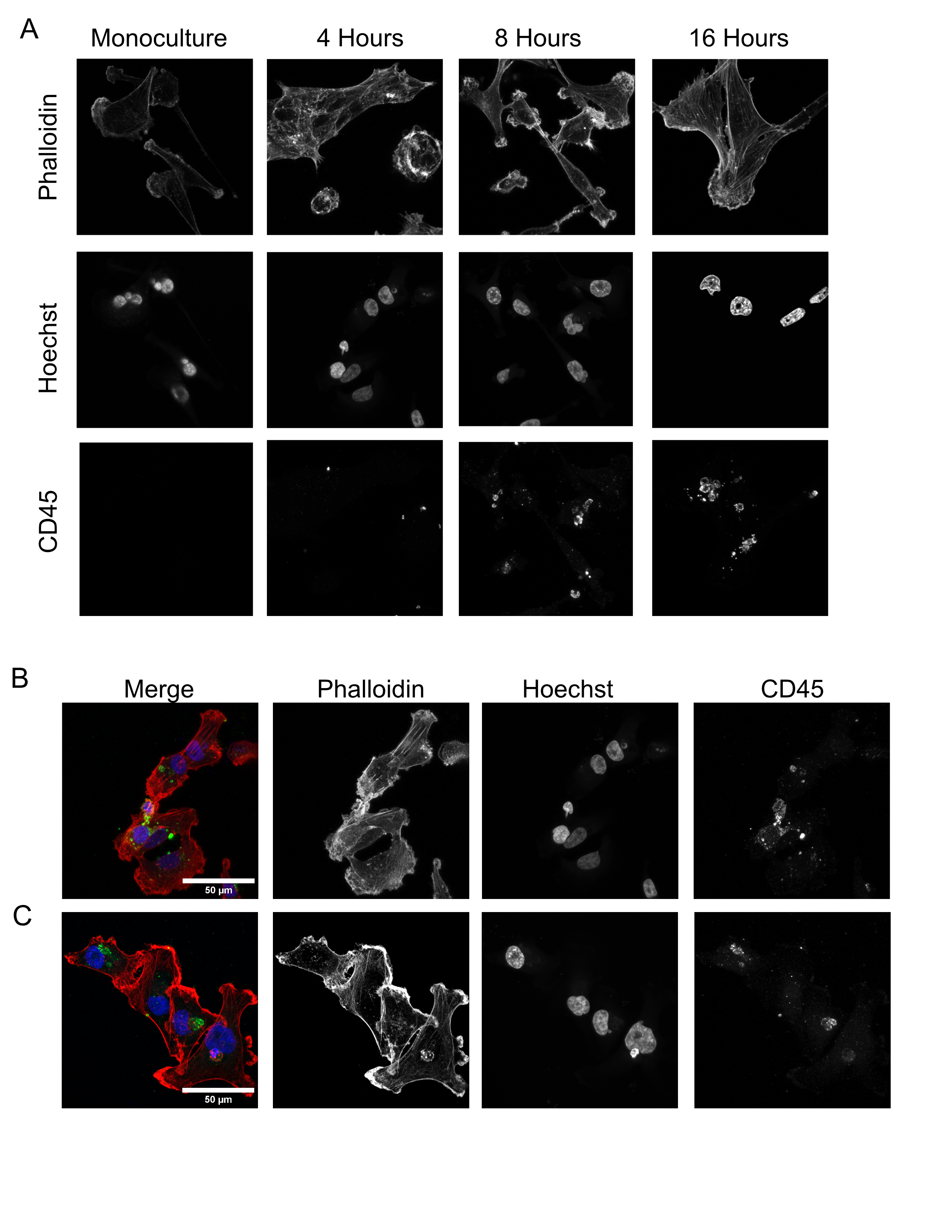

Supplement: Supplemental Information 3 — (A) Single channel images of co-cultured MDAMB231 shown in Fig. 3A. Additional merge and single channel images of trogosomes found present in (B) MDAMB231 and (C) MDAMB436 cells. [file peerj-13-19236-s003.png]

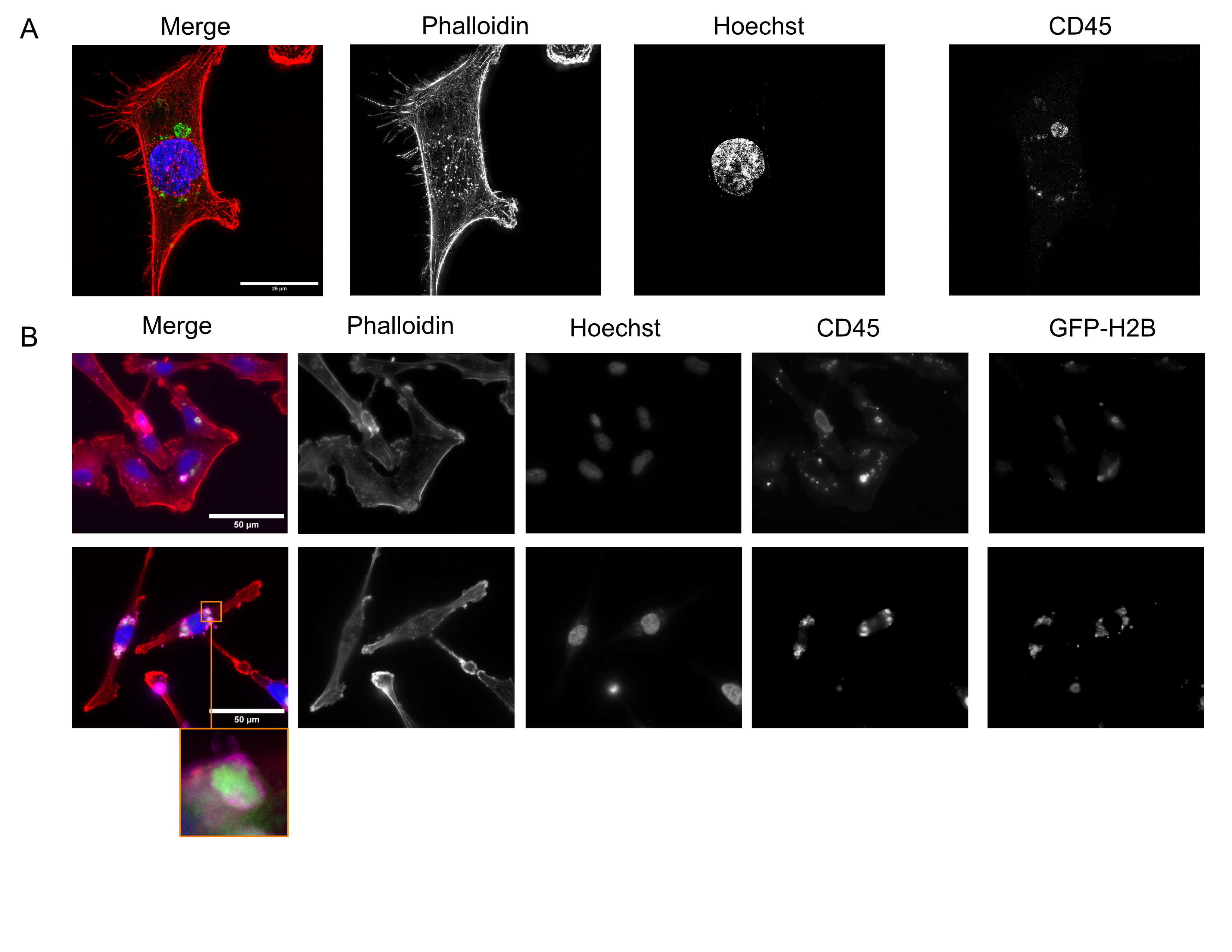

Supplement: Supplemental Information 4 — (A) SIM image of MDAMB231 cells containing a trogosome without positive Hoechst labeling. (B) Images of MDAMB231 cells containing GFP-H2B with merge images on the leftmost panel and single channel images in their respective columns. [file peerj-13-19236-s004.png]
